# Early detection of children at risk for antisocial behaviour using data from routine preventive child healthcare

**DOI:** 10.1186/1471-2431-12-24

**Published:** 2012-03-09

**Authors:** Sijmen A Reijneveld, Matty R Crone, Gea de Meer

**Affiliations:** 1 Department of Health Sciences, University Medical Center Groningen, University of Groningen, Groningen, The Netherlands; 2 TNO (Netherlands Organization of Applied Scientific Research), Quality of Life, Leiden, The Netherlands; 3 Department of Public Health and Primary Care, Leiden University Medical Center, Leiden, The Netherlands; 4 Municipal Health Service Fryslân, Leeuwarden, The Netherlands

**Keywords:** Antisocial behaviour, Early detection, Well-child care record, Prevention

## Abstract

**Background:**

Youth antisocial behaviour is highly prevalent. Young people are usually not willing to disclose such behaviour to professionals and parents. Our aim was to assess whether child health professionals (CHP) working in preventive child healthcare could identify pre-adolescents at risk for antisocial behaviour through using data that they obtain in routine practice.

**Methods:**

CHPs examined a national sample of 974 pre-adolescents aged 8-12 years (response 79.1%), and interviewed parents and children during routine well-child assessments. We obtained data on family background and current health of the child from the CHP; on developmental concerns from parents, and on social and emotional well-being, injuries, and substance use from the children. Antisocial behaviour concerned the adolescent-reported 15 item International Self-Reported Delinquency study questionnaire, among which are 5 items on violence against people.

**Results:**

The prevalence of 2+acts of any antisocial behaviour was 21.8%, and 33.9% for 1+acts of violence (10.5% for 2+). Children who were male, had a young mother, no parent employed, recent injuries, poor performance at school or who were bored by school, and who had parental concerns more often reported 2+antisocial acts and 1+violence against people. Detection algorithms on the basis of these variables were moderately able to classify outcomes, with Areas-Under-the-Curves ranging from 0.66 to 0.71.

**Conclusions:**

Data from routine well-child assessment can help CHPs to detect pre-adolescents at risk for antisocial behaviour, but detection algorithms need to be further improved. This could be done by obtaining additional information on factors that are associated with antisocial behaviour.

## Background

Youth antisocial behaviour is a major public health problem with detrimental effects for both victims and offenders [1-4]. Regarding victims, estimates in the year 2000 worldwide were that about 4400 people died each day because of intentional violence, and many thousands more suffered other health consequences [5,6]. These figures have probably risen since then [5]. Regarding the detrimental effects for the offender, early-onset antisocial behaviour has been shown to be associated with antisocial personality disorder, substance dependence, and depression [3,7-12].

Health gains appear to be greatest if the entire range of antisocial behaviour is addressed, including less severe variants. Apparently, addressing the most severely antisocial young people may yield gains for the group concerned [3,13,14], but antisocial behaviours such as fighting, setting fires, and theft are much more prevalent [4-6]. And these acts may have even greater effects on the community due to the resulting injuries and feelings of insecurity. In addition, health gains among these offenders may end up being much greater as well, due to the larger size of the group and to the fact that early stages of antisocial behaviour are more sensitive to modification.

Community paediatric services, such as those in the USA and the Netherlands, which offer routine preventive healthcare services to the entire population, are an ideal setting for the early detection of psychosocial problems, especially for antisocial behaviour, because interventions are available [15-17]. Early detection of behavioural problems is already a routine part of this preventive child healthcare program [18-20], but no evidence exists as to whether antisocial behaviour can really be detected. In particular, children are apparently unwilling to report their own antisocial behaviour to child health professionals (CHPs, doctors, and nurses). One alternative might be to identify antisocial children by other characteristics that are registered routinely during these preventive assessments. Evidence on the validity of this is lacking, however.

The aim of this study was to assess whether CHPs are able to identify pre-adolescents at risk for antisocial behaviour on the basis of data that they routinely collect in well-child practice.

## Methods

### Sample

We obtained a national sample using a two-stage selection procedure. In the first stage, a random sample of services addressing school-aged children was drawn using random numbers (15 out of a total of 40 services) [20,21]. The sample was stratified by region and degree of urbanization of the districts. In the second stage, each service provided a random sample of about 100 children for two age groups (5-6 years and 8-12 years), to the extent that they provided services for them. Moreover, children from two of the largest immigrant communities in the Netherlands, that is, children of Moroccan and Turkish origin, were oversampled by about one-third compared to their share in the population as registered in the municipal population registers; registration in these registers is obligatory for all residents of the Netherlands. This was done to enable the assessment of the quality of early identification of behavioural and emotional problems by PCH among children from these groups [20,21]. We only used the 11 services that served grades 6-8 (9-12 years) in the latter group, since children aged 8 were too young to fill out questionnaires without assistance. Out of these, 974 (79.1%) agreed to participate. Differences between responding and non-responding children regarding sex, age, ethnic background, and degree of urbanization were small according to Cohen's effect size, and the sample was representative for the Dutch population after weighting to adjust for the stratified sampling [22].

### Procedure and measurements

The data were collected in a standardized way as part of routine well-child assessments, similar to that of previously reported studies on the monitoring of child health [18,20,23]. These monitoring studies aimed at thousand respondents per age-group, which had been shown to generally suffice regarding the numbers as needed for monitoring purposes. Parents and children were mailed questionnaires, along with the routine invitation for the assessment. These were returned at the start of the visit in sealed envelopes. For the current study, only the child questionnaire was used. After each child's physical examination, the CHP obtained socio-demographic data and information from the mental/physical health history either from the child's health record or from a standardized interview with the parents. The design of the study was approved by the Institutional Review Board of the Leiden University Medical Center, including verbal consent. All questionnaires as used were piloted first. This led to the conclusion that the child questionnaire could not be filled out adequately by some children aged 8 years. Because of that we restricted that questionnaire to those aged 9 and over.

*Antisocial behaviour *was measured by the 15-item questionnaire of the International Self-Reported Delinquency study (ISRD; Additional file 1). The ISRD-study group defines antisocial behaviour as concerning both problem behaviour and youth-related offences. The questionnaire asks on antisocial acts regarding property (7 items), people (violence; 7 items), and police contacts (1 item) in the past 12 months (Additional file 1) [4,24]. Answering options were: "never," "once," "a couple of times," "often," and "very often," dichotomized as "never" vs. "often" (at least once). Out of these, we selected five items that concerned severe violence against people which would be most likely to reflect the effects of antisocial behaviour on the society [4,6].

*Additional child-reported measurements *concerned emotional well-being, friends, whether school was boring, school performance, recent injuries, and substance use (see Table 1).

**Table 1:** Data included in the study categorised by the likelihood of collection during routine well-child assessments

Commonly available	
Gender (PCH)	Male vs. female
Age (PCH)	8-10, 11-12 years
Ethnic background (PCH)	Dutch, Moroccan/Turkish, Surinamese/Antillean, other non-European
Number of siblings (PCH)	≥ 2 vs. < 2
Age of father, mother at birth of child (PCH)	≤ 26 years (young) vs. higher age
Educational level of father and mother (PCH)	≤ 12 years (low) vs. more (high/intermediate)
Urbanization (PCH)	City > 250,000 inhabitants (urban) vs. smaller
At least one parent having a paid job (PCH)	Yes vs. no
Family composition (PCH)	One parent vs. two parents
Chronic disease of child (PCH)	Yes vs. no
Parental concerns (on parenting in general; child development, behaviour, emotions, social functioning, and/or academic performance) (P)	≥ 1 vs. none
Life event in the past 12 months (moved to a new house, new sibling, parental divorce, parent unemployed, death or severe disease of a household member, severe disease of the child) (P)	≥ 1 vs. none
Under treatment for psychosocial problems (PCH)	Yes vs. no
Medical treatment for injuries in the past 12 months (C)	≥ 2 times vs. < 2 times
**Likely available**	
School performance compared to classmates (C)	Poorer vs. equal to/better
Feeling bored at school (C)	(Very) often vs. sometimes/never
Appreciation of life (C)	< 7 vs. ≥ 7 on a 10-point scale (1 = negative,10 = highly positive)
Having sufficient friends (C)	Yes vs. no
Appreciation of school (C)	Dislike (very) much vs. like (very) much
**Possibly available**	
Whether substances were ever used (alcohol, cigarettes) (C)	Yes vs. no

(P) parents (C) child (PCH) Preventive Child Healthcare record, verified by asking parent

*The socio-demographic variables *assessed were: sex, age, ethnicity, family composition, siblings living in the family at the time of the study, parental educational level, income and employment status, and the agglomeration's degree of urbanization. Ethnic background was assessed by country of birth of the child's parents. On the basis of the migration histories of various groups living in the Netherlands, this was coded as: Dutch-born; from a (former) Dutch colony (at least one parent born in Surinam or the Dutch Antilles); from countries in which Dutch employers recruited unskilled labourers in the 1960s and 1970s ("labour immigrant," at least one parent born in Turkey or Morocco); other industrialized countries (i.e., member states of the Organization for Economic Co-operation and Development); and other non-industrialized countries [20]. Parental educational level was defined as the number of years of education needed to obtain the highest degree completed by the parent concerned. Family composition was defined as the number of biological parents that were part of the family in which the child lived. Urbanization was defined as one of the four big cities (> 250,000 inhabitants) vs. smaller agglomerations.

*Mental/physical health history *was defined as life events during the past 12 months (moved to a new house, new sibling, parental divorce, parent unemployed, death or severe disease of a household member, severe disease of the child), child under treatment because of psychosocial problems, and parental concerns (about child rearing in general, development, behaviour, emotions, social functioning, academic performance).

Data from the well-child visits were categorized on the basis of their likelihood of being routinely available at the time of such visits as "commonly," "+likely," and "+possibly available" (Table 1). Information from the first category can routinely be provided by either the parent or the child's health record. For the second one, the child is needed as informant. And for the third one, this information can only be obtained in a valid way if confidentiality is guaranteed. Categories and sources for all variables are presented in Table 1.

### Statistical analyses

Statistical procedures were performed using the SPSS 16.0 statistical software package. First, we developed a detection algorithm for any antisocial behaviour (out of all 15 items at least two, to exclude incidental occasions) and for severe violence against people (at least one out of the 5 items) using data from routine well-child visits. Variables were selected consecutively from the three categories ("commonly," "+likely," and "+possibly available," Table 1) using logistic regression analysis with forward selection at p < 0.05 per step. Apparently, routine preventive child healthcare does not collect information on all established predictors of antisocial behaviour in adolescents. This set of variables was thus restricted by current practices in preventive child healthcare.

Next, detection algorithms for use in CHP practice were constructed based on the final models for each step. These algorithms consisted of weight derived from the odds ratios of all variables in the model. For each child, the detection score was calculated as the sum of the variables' weights that determined the detection algorithm. We then computed Areas-Under- the-Receiver-Operator-Curve (AUC) as a measurement of the ability of the algorithm to discriminate between children with and without antisocial behaviour. AUCs can range from 0.5 (no discrimination) to 1.0 (perfect discrimination), and a value > 0.7 is preferred for diagnostic procedures [25].

## Results

Of the 974 children, 21.8% reported having been involved in at least two antisocial acts (Table 2); the most prevalent kind was threatening to hit someone. And 33.9% reported at least one act of severe violence against people.

**Table 2:** Antisocial acts in the past 12 months, N = 974

	N	(%)
**Property**		
Shoplifting	41	(4.2)
Damaging public property	34	(3.5)
Damaging something on the street	27	(2.8)
Setting something on fire	19	(2.0)
Theft at school	38	(3.9)
Theft at home	42	(4.3)
Entering a place in order to steal	4	(0.4)
**Violence against people**		
Threatening someone with a knife or other weapon *(severe)*	43	(4.4)
Forcing someone to hand over money or valuables *(severe)*	16	(1.7)
Quarrelling with a teacher	147	(15.1)
Insulting a teacher at school	52	(5.4)
Hitting or kicking a parent/caregiver *(severe)*	56	(5.8)
Telling someone you will beat him/her up *(severe)*	215	(22.1)
Beating someone up, not out of self-defence *(severe)*	127	(13.1)
**General**		
Interrogated as a suspect by the police	31	(3.2)
**Total**		
≥ 1 acts	442	(45.4)
≥ 2 acts	212	(21.8)
≥ 3 acts	116	(11.9)
*Of which severe violence*		
*≥ 1 acts*	*330*	*(33.9)*
*≥ 2 acts*	*102*	*(10.5)*
*≥ 3 acts*	*23*	*(2.4)*

Table 3 shows the results of logistic regression analyses aimed at the selection of variables for the detection algorithm based upon "commonly" available data from routine well-child assessments (Table 1). Children that confirmed *any act of severe violence against people in the past 12 months *were more frequently (p < 0.05) male, of labour-immigrant descent, with a relatively young father or mother, were more frequently under treatment, and they more frequently had had injuries requiring treatment. Their parents were more frequently unemployed, and had parenting concerns more frequently.

**Table 3:** Prediction of child-reported violence against people and at least two antisocial activities from data obtained during well-child visits: odds ratios (95% confidence intervals)

	Any severe violence against people		At least 2 cases of any antisocial activity	
	Unadjusted model	Reduced model	Unadjusted model	Reduced model
**Commonly available**				
Male vs. female gender	**2.14 (1.62; 2.82)**	**2.09 (1.56; 2.79)**	**2.46 (1.78; 3.41)**	**2.27 (1.62; 3.18)**
Age 10-11 vs. 8-10	0.99(0.75; 1.32)		1.25 (0.90; 1.75)	
Ethnic background				
Dutch	1.00 (reference)		1.00 (reference)	
Surinamese/Antillean ('former colony')	1.17 (0.64; 2.15)		0.76 (0.35; 1.66)	
Moroccan/Turkish ('labour immigrants')	**2.14 (1.47; 3.12)**		**2.00 (1.33; 3.01)**	
Other non-industrialized	1.25 (0.68; 2.30)		1.19 (0.59; 2.40)	
Two siblings or more, N = 982	1.02 (0.78; 1.35)		1.26 (0.92; 1.72)	
Mother's age at childbirth < 27 years vs. 27+	**1.66 (1.22; 2.26)**	**1.65 (1.19; 2.27)**	**1.62 (1.15; 2.28)**	**1.47 (1.02; 2.12)**
Father's age at childbirth < 27 years vs. 27+,	**1.55 (1.01; 2.38)**		1.15 (0.70; 1.88)	
Mother's education, low vs. high/intermediate	1.21 (0.92; 1.59)		**1.42 (1.04; 1.94)**	
Father's education, low vs. high/intermediate	**1.58 (1.20; 2.07)**		**1.61 (1.17; 2.20)**	**1.49 (1.07; 2.08)**
Region urban vs. non-urban	1.17 (0.87; 1.57)		1.35 (0.97; 1.88)	
No parent employed vs. at least one	**2.61 (1.49; 4.57)**	**2.62 (1.47; 4.66)**	**2.33 (1.30; 4.18)**	
One-parent family vs. other	1.16 (0.74; 1.82)		1.38 (0.84; 2.24)	
Chronic disease of the child, yes vs. no	1.39 (0.94; 2.06)		**1.72 (1.14; 2.61)**	
Parental concerns about the child	**1.86 (1.41; 2.45)**	**1.59 (1.19; 2.12)**	**2.29 (1.69; 3.14)**	**1.91 (1.37; 2.67)**
Life events in the past 12 months	0.99 (0.75; 1.30)		1.12 (0.82; 1.54)	
Under psychosocial treatment	**1.92 (1.12; 3.31)**		**2.92 (1.68; 5.08)**	**1.93 (1.06; 3.48)**
1+ Injuries during past 12 months vs. none	**2.46 (1.37; 4.42)**	**2.31 (1.24; 4.29)**	**2.26 (1.23; 4.15)**	**1.97 (1.04; 3.75)**
**Likely available**				
School performance, mean/lower vs. good	**1.76 (1.29; 2.42)**	**1.53 (1.09; 2.15)**	**1.71 (1.21; 2.43)**	
Bored at school, yes vs. no	**1.90 (1.07; 3.36)**	**1.69 (1.12; 2.55)**	**2.32 (1.28; 4.20)**	
Well-being, 6 or less vs. 7+	**1.76 (1.11; 2.78)**		**2.30 (1.42; 3.72)**	**1.83 (1.10; 3.03)**
Sufficient friends, no vs. yes	**1.93 (1.19; 3.12)**		1.50 (0.88; 2.55)	
Likes school, no vs. yes	**2.01 (1.36; 2.96)**		**2.11 (1.39; 3.20)**	**1.64 (1.05; 2.56)**
**Possibly available**				
Whether substances were ever used	**1.97 (1.32; 2.95)**		**2.44 (1.60; 3.73)**	**2.08 (1.33; 3.26)**

The final reduced multivariable regression model included five variables with p < 0.05 that determined the detection algorithm, that is, male gender, having a young mother, no employed parent, parental parenting concerns, and being treated for injuries in the past 12 months. Out of the data from the child, two additional ones were selected in the multivariable regression model: self-reported school performance and considering school to be boring. Finally, regarding data possibly available, substance use was not selected for the multivariable model.

Regarding *at least two out of 15 antisocial acts*, mostly similar variables were selected, both univariable and multivariable. From the variables likely available, the only difference was that paternal educational level was selected instead of parental employment status. From the data likely available, overall well-being and assessment of school were selected, instead of self-rated school performance and boring at school (Table 3). Finally, regarding data possibly available substance use was selected for the multivariable model.

Based on odds ratios, we constructed Receiver Operator Curves for each algorithm and assessed the AUCs in order to evaluate the discriminatory performance of each algorithm. For the initial detection algorithm based upon data commonly available, the AUCs (95% confidence intervals (CI)) were 0.66 (0.63; 0.70), and 0.69 (0.65; 0.73) for any severe violence against people and at least two antisocial acts, respectively (Table 4). After inclusion of all variables, these AUCs increased slightly; see Table 4. The AUCs were generally in a moderate range.

**Table 4:** Performance of detection algorithms on antisocial behaviour: Areas under the Curve and 95% confidence intervals

Groups of predictors	Severe violence against people	At least two antisocial acts
Commonly available	0.66 (0.63; 0.70)	0.69 (0.65; 0.73)
Likely available	0.68 (0.64; 0.72)	0.70 (0.66; 0.74)
Possibly available	(no addition)	0.71 (0.67; 0.75)

## Discussion

This study was the first to develop an algorithm for the detection of antisocial behaviour in 8-12 year-old primary school children, using information that can be obtained during well-child visits. Our findings show that this information may indeed help CHPs to identify children who are at increased risk of antisocial activities, in general, and violence against people, in particular. However, the predictive power of the detection algorithms as measured by the AUC was relatively poor.

Our findings show that a detection algorithm based on routinely available data may be a useful first step in a multi-step detection procedure in CHP practice. In a second step, confirmative testing on antisocial acts would be needed in a selected part of the population. The resulting final group could be offered further early intervention which has been shown to decrease antisocial behaviour in about 66% [15-17]. This could yield a 20-29% reduction in antisocial behaviour in the community, albeit at the expense of a group which had been false-positive at earlier stages of the procedure.

The discriminatory power of the detection algorithm is moderate, which indicates that it needs to be improved for application in routine preventive child healthcare. Several approaches may yield such an improvement. First, other characteristics might be included in the detection algorithm. As reported by others, antisocial behaviour was associated with male gender, large family, young mother, poor child-parent relationship, and substance use [8,26-29]. One might consider to extend the preventive child healthcare assessment procedure by other potential predictors. Candidates might be child characteristics such as (low) intelligence or academic performance, externalizing behaviour, hyperactivity and behavioural problems; parent characteristics such as (poor) parental supervision, hostile parenting, physical punishment, parent-child separation, deviant mother-child interactions, parental criminality, maternal smoking during pregnancy, and family psychiatric history [30-32]; and social factors, such as antisocial peers and high delinquency neighbourhood [32]. If these factors would be included in the assessment procedure, it definitely requires additional study whether this could be managed in the available time per visit. In addition, it requires additional study whether data in the 'possibly available' category, i.e. substance use, can be obtained in a valid way indeed.

As a second means to improve detection, one might consider having the child fill out the same questionnaire as used in this study or a similar one. However, if the child would have to give the completed questionnaire to the CHP, this is very likely to lead to biased information compared to the setting of this study in which confidentiality was guaranteed to the child with only the researchers reading the answers after removal of all identifying data.

Third, information from teachers might be added. Petras et al. found good prediction of violence using the Teacher Observation of Classroom Adaptation (TOCA) [13,14]. However, consent needs to be obtained from parents if applied for well-child purposes, which may limit its applicability.

Fourth, parent-reported questionnaires on antisocial behaviour might be added to the routine behavioural assessment at the time of well-child visits. However, it may in fact be quite questionable whether the parent would actually be well-informed about any such behaviour, even in cases of great concern.

We defined antisocial behaviour as an act of violence against either property or people [4,24], leading to direct and indirect effects on health [5,6]. This broad definition may explain the higher prevalence of antisocial behaviour in our study compared with previous studies that used a more restrictive definition based upon judicial prosecution [13,14], or psychopathology [8,26]. We think that our definition better reflects antisocial behaviour as perceived in the community, given the process of development of the ISRD questionnaire [4,24]. Our definition may include transient antisocial behaviour, but early onset has been shown to be predictive for a life-long career of such behaviour. Early detection and intervention may turn the trait into a socially acceptable lifestyle [1-4]. Future studies are needed to evaluate the effectiveness of intervention in antisocial behaviour after detection based on information obtained from routine well-child assessments or school health records. Finally, one might challenge our definition of violence against people, in particular the inclusion of threatening someone. Repeating the analyses with exclusion of this item did not affect the results, however.

### Strengths and limitations

The strengths of this study lie in its community-based setting using information that is commonly available from school health records to detect pre-adolescents at risk of antisocial behaviour. The limitations of our study involved missing data, the data collection procedure, and the definition of antisocial behaviour. First, we measured anti-social behaviour using self-report. This may have resulted in underreporting. However, the alternatives - observation and proxy-reporting - would likely yield much more underreporting, and previous studies have shown the ISRD questionnaire to be highly valid [4,24]. In addition, the data might have been collected in a more rigorous way than would actually occur in routine well-child care. Therefore, our results need confirmation in routine practice.

### Implications

Our findings imply that well-child health care can support the early detection of antisocial behaviour. Additional measurements on other predictors of antisocial behaviour are needed, however, to further strengthen the subsequent stages of this early detection. This could in the end contribute towards resolving what is a major threat to both the health of the individuals involved and to society as a whole.

## Conclusions

We conclude that data from routine well-child assessment may help child health professionals to detect pre-adolescents at risk for antisocial behaviour, but that detection algorithms need to be further improved.

## Abbreviations

CHP: Child health professional; ROC: Receiver-operating-characteristics; AUC: Area under the ROC-curve; 95% CI: 95% confidence interval.

## Competing interests

The authors declare that they have no competing interests.

## Authors' contributions

SAR was the principal investigator of this study, wrote the study protocol, and wrote the paper. MRC supervised the data collection for the study. SAR and GdM did the statistical analyses GdM contributed to important parts of the text All authors discussed the protocol, formulated the final design, discussed the results of the statistical analyses, discussed the texts, and read and approved the final manuscript.

## Pre-publication history

The pre-publication history for this paper can be accessed here:

http://www.biomedcentral.com/1471-2431/12/24/prepub

## Supplementary Material

Additional file 1 **Questionnaire on antisocial acts in the past 12 months as completed by the children**. Click here for file
