# Supplementary material for: Early detection of children at risk for antisocial behaviour using data from routine preventive child healthcare
Source: BMC Pediatr. 2012 Mar 9;12:24. doi: 10.1186/1471-2431-12-24 (PMC3314552; doi:10.1186/1471-2431-12-24)
Supplement: Additional file 1 — Questionnaire on antisocial acts in the past 12 months as completed by the children. [file 1471-2431-12-24-S1.DOC]

# Appendix

## Questionnaire on antisocial acts in the past 12 months as completed by the children.

| How often have you been involved in the following in the past 12 months? |
| --- |
| 1. Taking something from a shop or department store without paying for it? |
| 1. Vandalizing walls or public property such as bus stops or bins with graffiti? |
| 1. Damaging something in the street on purpose; for example a traffic sign, a street lamp, a public telephone, a bus stop, or a waste bin? |
| 1. Setting something on fire on purpose that was not yours (e.g., a shed, a cellar, a waste bin, or something else)? |
| 1. Stealing something from classmates, teachers, or other people at school? |
| 1. Stealing money from home (e.g., from your father or mother)? |
| 1. Breaking into a building without permission, for example, a school or a house, for the purpose of stealing something? |
| 1. Threatening someone with a knife or another weapon (e.g., a stick) just to frighten him/her? * |
| 1. Forcing someone to give you money or valuable things (that were not yours)? * |
| 1. Quarreling with a teacher at school? |
| 1. Insulting a teacher at school? |
| 1. Hitting or kicking one of your parents/caregivers? * |
| 1. Telling someone you would beat him/her up? * |
| 1. Beating someone up? (Hitting or kicking back in self-defence does not count) * |
| 1. Being interrogated by the police because you were suspected of something that was illegal? |

* Severe violence against people
